# Evaluating the impact of evidence-based nursing in combination with clinical nursing pathway for nursing care of patients with stroke: A protocol for systematic review and meta-analysis

**DOI:** 10.1097/MD.0000000000028278

**Published:** 2022-01-14

**Authors:** Shao-Yan Ma

**Affiliations:** Department of Neurology, Wuhan Asia General Hospital, Wuhan, Hubei, China.

**Keywords:** clinical nursing, evidence-based nursing, meta, nursing care, stroke

## Abstract

**Objectives:**

Strokes are among the leading conditions that lead to disability and death. Currently, there is a lack of ideal nursing care for stroke patients. The aim of this study is to assess the effect of combining evidence-based nursing and clinical nursing pathway to establish a nursing model to provide care for patients who suffered a stroke.

**Methods:**

A comprehensive search of online-based databases will be carried out to identify relevant publications, the databases include EMBASE, Cochrane Library, PubMed, VIP databases, Chinese National Knowledge Infrastructure, and WanFang database. The search will consider all Randomized Controlled Trials, interrupted time series studies, and controlled before and after studies, all related to providing care for neurology and strokes by combining evidence-based nursing and clinical nursing pathway to elevate access and outcomes for patients with stroke. The language of publications will be restricted to English and Chinese. The author will select studies, extract data, and evaluate the quality of the involved articles. RevMan 5.3 software will be employed to perform all statistical analysis.

**Results:**

The outcomes of the proposed study will provide scientific evidence for the nursing care of evidence-based nursing combined with clinical nursing pathway for stroke patients.

**Conclusion:**

The planned systematic analysis will be first to examine the effect of combining evidence-based nursing and clinical nursing pathway to present a nursing model to treat stroke patients.

**Registration number:**

November 16, 2021.osf.io/6zr5a/. (https://osf.io/6zr5a/).

## 1 Introduction

Strokes are a common cause of brain damage. When a person gets a stroke, the blood supply to the brain is interrupted, which causes sudden, severe damage to part of the brain because of infarction (embolus or thrombus) or haemorrhage (subarachnoid or cerebral). Subsequently, the damaged part of the brain loses functionality, and the degree of the damage is dependent on the lesion's size and site. Generally, strokes cause weakness, hemiplegia, perceptual dysfunction, loss of control of the bladder and bowel, and disruption of vision and/or speech.^[1]^

Globally, strokes are the second leading factor leading to fatalities and morbidity, and the prevalence of strokes are increasing as the global population is ageing.^[2]^ Indeed, 80% of patients who survive the acute phase of stroke are discharged after their condition stabilizes. Moreover, 77% of the patients have hemiplegia, and 28.6% suffer dysfunctions related to language and other conditions, and these complications inhibit the mobility of stroke patients and their overall life standard.^[3–5]^ Thus, establishing superior methodological scientifically proved effective nursing for those with hemiplegia after a stroke is critical to minimize the occurrence of complications and elevate the life standard of patients.

In modern society, there is an increasing demand for high-quality medical services and standards. The traditional routine nursing mode is obsolete when it comes to addressing the increasing demands from patients.^[6]^ Clinical practitioners recognize and propose the adoption of a high-quality nursing model to elevate the life standards of patients through satisfactory nursing care.^[7]^ Evidence-based nursing is a novel nursing approach that considers the explicit state and requirements of patients. Accordingly, clinical evidence is collected by referring literature to frame a feasible scientific nursing strategy, and make timely adjustments to achieve the best nursing effect through nursing measures.^[8]^ As a fundamental nursing practice, evidence-based nursing is difficult to apply. However, the effect of combining clinical nursing pathway and evidence-based nursing to provide nursing care for stroke patients is not known. Thus, the present study will be conducted to systematically assess the effect of combing evidence-based nursing and clinical nursing pathway to offer nursing treatment for patients who underwent a stroke.

## 2 Objectives

The proposed meta-analysis will summarize past studies to assess the effect of the combination of evidence-based nursing and clinical nursing pathway to offer nursing services for stroke patients.

## 3 Methods

The present study will be carried according to the preferred reporting items for systematic reviews and meta-analyses statement.^[9]^ In addition, the study is registered under the Open Science Framework (Registration number: DOI 10.17605/OSF.IO/6ZR5A. Available from: https://osf.io/6zr5a/).

### 3.1 Ethics and dissemination

This study will not require an ethical approval.

### 3.2 Eligibility criteria for included studies

#### 3.2.1 Types of studies

All Randomized Controlled Trials, controlled before and after studies, and interrupted time series studies of neurology and strokes associated with combining evidence-based nursing with the clinical nursing pathway to increase access and improve outcomes for patients with stroke will be included. Other forms of studies will not be considered, including letters, case reports, conferences, and reviews.

#### 3.2.2 Types of participants

All participants diagnosed with stroke will be included without any constraints on nationality and gender.

#### 3.2.3 Types of interventions

The participants in the empirical group must be administered the combination of evidence-based nursing and clinical nursing pathway. The interventions of the control group can include any nursing intervention or no nursing intervention.

#### 3.2.4 Types of outcomes

Outcomes include Barthel Index, Fugl-Meyer motor score, Self-Rating Depression Scale, Self-Rating Anxiety Scale, National Institute of Health stroke scale, and Mini-Mental State Examination.^[10–15]^

### 3.3 Search methods for the identification of studies

#### 3.3.1 Electronic searches

An inclusive search of electronic databases from will be conducted from their inception until November 3, 2021. The databases include PubMed, Cochrane Library, Chinese National Knowledge Infrastructure, EMBASE, VIP databases, and WanFang database. The language of the publications will be restricted to English and Chinese. The search process will be based on the following keywords: stroke, evidence-based nursing, clinical nursing pathway, and randomized controlled trials.

#### 3.3.2 Search other sources

The authors will manually search the lists of references in studies obtained from the search to identify every source of grey literature. In addition, the author will also perform a search on Google Scholar to include all related articles available on the Internet.

### 3.4 Data collection and analysis

#### 3.4.1 Study selection

The authors will assess the title and abstract of each potential study. The process of screening the studies will follow the pre-defined criteria using a standardized form. The study selection process will follow the PRISMA flow chart. The authors will consult a senior researcher to sort out any disagreement that occurs during the process.

#### 3.4.2 Data extraction

Based on the search strategy mentioned above, an investigator will screen the titles and abstracts of all potentially suitable articles. Next, the reviewer will gather the following information from the selected studies: study design, inclusion criteria, intervention method, basic characteristics of included studies, outcome measures, reported adverse events. All discrepancies shall be resolved by consulting with a senior researcher.

#### 3.4.3 Methodological quality assessment

The methodological value of each considered study will be assessed using the Cochrane bias risk evaluation tool.^[16]^ There are 7 items in the bias risk tool, and in each item, the assessment is rated as low bias risk, unclear bias risk, and high bias risk. The bias risk in each study selected for inclusion will be assessed by 2 independent reviewers. A senior researcher will be consulted to resolve any discrepancies.

#### 3.4.4 Measures of treatment effect

The continuous outcome data is presented as mean differences or standardized mean differences together with 95% Confidence Intervals. The dichotomous outcome data is presented as relative risks with 95% confidence interval.

#### 3.4.5 Dealing with missing data

Partially complete or non-reported data will be obtained by reaching out to the corresponding author via email. In instances where the studies have partial data, the respective studies shall be omitted.

#### 3.4.6 Assessment of heterogeneity

A statistically significant heterogeneity is assumed when the *P* value based on the Chi-Squared test is less than 0.10 or *I*^*2*^ is over 50%, accordingly, the random-effects will be used for the pooled data. A two-sided *P* value less than .50 will be regarded as statistically significant.

#### 3.4.7 Sensitivity analysis

The one-by-one elimination method will be employed to perform a sensitivity analysis to assess the stability of the results of the meta-analysis.

#### 3.4.8 Subgroup analysis

A subgroup analysis will be conducted on the basis of the age and the type of nursing intervention.

#### 3.4.9 Assessment of reporting biases

Funnel plots will be employed to evaluate the publication biases, if applicable.

## 4 Discussion

The planned systematic review and meta-analysis aims to summarize the most recent literature to assess the effect of combining clinical nursing pathway with evidence-based nursing to offer nursing care for stroke patients. The findings of the study will report comprehensive, reliable, and updated evidence to ascertain the potential of clinical nursing pathway combined with evidence-based nursing. Moreover, the outcomes are a useful reference for practitioners implementing nursing intervention and collecting data from stroke patients. In addition, scholars and policymakers can also refer to the outcomes of the study. Implementing nursing methods to minimize the occurrence of complications after a stroke is crucial to elevate the life standard of patients. Thus, the findings of the review will provide scientific evidence for high-quality nursing for stroke patients with hemiplegia.

## Author contributions

**Conceptualization:** Shao-Yan Ma.

**Data curation:** Shao-Yan Ma.

**Formal analysis:** Shao-Yan Ma.

**Funding acquisition:** Shao-Yan Ma.

**Investigation:** Shao-Yan Ma.

**Methodology:** Shao-Yan Ma.

**Project administration:** Shao-Yan Ma.

**Software:** Shao-Yan Ma.

**Validation:** Shao-Yan Ma.

**Writing – original draft:** Shao-Yan Ma.

**Writing – review & editing:** Shao-Yan Ma.
